# DWNN-RLS: regularized least squares method for predicting circRNA-disease associations

**DOI:** 10.1186/s12859-018-2522-6

**Published:** 2018-12-31

**Authors:** Cheng Yan, Jianxin Wang, Fang-Xiang Wu

**Affiliations:** 1 School of Information Science and Engineering, Central South University, 932 South Lushan Rd, ChangSha, 410083 China; 2 School of Computer and Information,Qiannan Normal University for Nationalities, Longshan Road, DuYun, 558000 China; 3 Biomedical Engineering and Department of Mechanical Engineering, University of Saskatchewan, Saskatoon, SKS7N5A9 Canada

**Keywords:** CircRNA, CircRNA-disease association, Gaussian interaction profile, Kron-RLS

## Abstract

**Background:**

Many evidences have demonstrated that circRNAs (circular RNA) play important roles in controlling gene expression of human, mouse and nematode. More importantly, circRNAs are also involved in many diseases through fine tuning of post-transcriptional gene expression by sequestering the miRNAs which associate with diseases. Therefore, identifying the circRNA-disease associations is very appealing to comprehensively understand the mechanism, treatment and diagnose of diseases, yet challenging. As the complex mechanism between circRNAs and diseases, wet-lab experiments are expensive and time-consuming to discover novel circRNA-disease associations. Therefore, it is of dire need to employ the computational methods to discover novel circRNA-disease associations.

**Result:**

In this study, we develop a method (DWNN-RLS) to predict circRNA-disease associations based on Regularized Least Squares of Kronecker product kernel. The similarity of circRNAs is computed from the Gaussian Interaction Profile(GIP) based on known circRNA-disease associations. In addition, the similarity of diseases is integrated by the mean of GIP similarity and sematic similarity which is computed by the direct acyclic graph (DAG) representation of diseases. The kernels of circRNA-disease pairs are constructed from the Kronecker product of the kernels of circRNAs and diseases. DWNN (decreasing weight k-nearest neighbor) method is adopted to calculate the initial relational score for new circRNAs and diseases. The Kronecker product kernel based regularised least squares approach is used to predict new circRNA-disease associations. We adopt 5-fold cross validation (5CV), 10-fold cross validation (10CV) and leave one out cross validation (LOOCV) to assess the prediction performance of our method, and compare it with other six competing methods (RLS-avg, RLS-Kron, NetLapRLS, KATZ, NBI, WP).

**Conlusion:**

The experiment results show that DWNN-RLS reaches the AUC values of 0.8854, 0.9205 and 0.9701 in 5CV, 10CV and LOOCV, respectively, which illustrates that DWNN-RLS is superior to the competing methods RLS-avg, RLS-Kron, NetLapRLS, KATZ, NBI, WP. In addition, case studies also show that DWNN-RLS is an effective method to predict new circRNA-disease associations.

## Background

Circular RNAs (circRNAs) are a class of endogenous noncoding RNAs with distinct properties and diverse cellular functions, unlike the linear RNAs with 5’ and 3’ termini which reflect start and stop of the RNA polymerase on the DNA template, and are generated by back splicing (3’-5’) or lariat introns [1–4]. The circRNAs are not easy to be degraded by exoribonucleases because they lack free ends [5, 6]. As forming a circRNA is usually considered a rare event in cells, it was suggested that they may be considered errors of normal splicing process [4, 7]. Therefore, despite their existence in both unicellular and multicellular organisms, they have been previously even disregarded as transcriptional noise or artifacts [8]. Nevertheless, with the advances of high-throughput deep sequencing and functional genomics, the knowledge of circRNAs has recently been learned substantially [9, 10].

To date, circRNAs have been found in various tissues and cell lines of plants, animals and so on [4, 11, 12]. Some circRNAs can be translated in some tissues or translated into a protein under splicing-dependent, cap-independent manner or other certain conditions [13]. Furthermore, circRNAs are expected to have other functions independent of their host genes because they have much longer half-life than other linear RNA transcripts [10]. Many circRNAs can regulate gene expression because they have strong potential to act as miRNA sponges or decoys [14]. In addition, some circRNAs can also function as protein sponges or decoys, and the best example is that protein MBL is prevented to bind to other targets when being tethered to a circRNA [15]. CircRNA circFoxo3 can also act as a protein scaffold, which binds to sites for mouse MDM2 and p53 [16]. Unlike the above functions of circRNAs are based on the fact that they are located to the cytoplasm, some circRNAs such as exon-intron circRNAs are retained in the nucleus and they may promote with transcription [17].

Through the understanding of functions of circRNAs, many evidences have shown that circRNAs play an important role in occurrence of human complex diseases, such as cancer [18]. CircRNA ciRS-7 has significant implications for diseases through efficiently regulating the activity of miRNA miR-7 [19]. Likewise, by sponging the miR-7, miR-17 and miR-214, cir-ITCH can increase the level of ITCH which further inhibits the Wnt pathway that is frequently aberrant in cancers [20, 21]. SRY can affect the proliferation, migration and invasion of cholangiocarcinoma cells, which is the sponge of miR-138 and can strongly suppress its level [22, 23]. CircRNA-MYLK level is elevated and correlated with BC (bladder carcinoma) progression and plays an oncogenic role in BC in vitro and vivo [24]. Circ-Foxo3 was minimally expressed in patient tumor samples and in a panel of cancer cells and its expression was found to be significantly increased during the cancer cell apoptosis [16, 25]. Circular RNA MTO1 can suppress hepatocellular carcinoma progression by acting as the sponge of miR-9 [26]. In addition, the aberrant expression of circCCDC66 also is associated with a late-stage diagnosis and metastases [27].

In recent years, some databases about circRNAs have been developed to further study the function mechanism of circRNAs. CircBase is the first database about circRNAs, which merges and unifies data sets of circRNAs and provides the interface to access, download, and browse the evidence supporting their expression within the genomic context [28]. CircRNADb is a comprehensively annotated human circular RNAs database, which containes 32,914 human exonic circRNAs from diversified sources and provides the genomic information, exon splicing, genome sequence, internal ribosome entry site (IRES), open reading frame (ORF) and references of these circRNAs [29]. PlantcircBase is a database of plant circRNAs, which also provided other functions such as visualization of the structures of circRNA based on their genomic position [12]. Likewise, PlantCircNet also is a database of plant circRNAs, which has the main feature of plantCircNet to provide visualized plant circRNA-miRNA-mRNA regulatory networks and can identify metabolic effects of circRNAs [30]. ExoRBase is a web-accessible database, which provides the circRNA, lncRNA and mRNA information by RNA-seq data analyses of human blood exosomes [31]. CircNet provides tissue-specific circRNA expression profiles and circRNA-miRNA-gene regulatory networks by utilizing sequencing datasets to systematically identify the expression of circRNAs in RNA-seq samples [32]. TSTD also provides the tissue-specific circRNAs and further characterizes the functions of these circRNAs [33]. The cancer somatic mutations that alter miRNA targeting and functioning are provided by SomamiR 2.0 database which also collects the associations between miRNA and other competing endogenous RNAs such as mRNAs, circRNAs and lncRNAs [34]. The CSCD is also a cancer-specific circRNAs database which identifies the cancer-specific circRNAs by analyzed the RNA-seq samples and further predicts the miRNA response element sites and RNA binding protein sites of each circRNA [35]. Circ2Traits is the circRNA-disease associations database, which is constructed by circRNA-miRNA associations, miRNA-disease associations and disease-SNPS associations [18]. To our knowledge, CircR2Disease is the first manually curated database about circRNA-disease associations by reviewing existing literatures and provides the important foundation to study the associations of circRNAs and diseases [36].

In general, we have obtained some significant progresses in understanding features and functions of circRNAs. In addition, some databases about circRNAs have also been constructed. However, current studies of circRNA-disease associations mainly focus on biomedical experimentations that are notoriously expensive and time-consuming. Therefore, there is a very urgent need to predict circRNA-disease associations by computational methods. To our knowledge, the development of computational approach is very limited because the databases of circRNA-disease associations are incomplete. However, circR2Disease provides the chance to effectively predict novel circRNA-disease associations through developing computational methods.

In this study, we develop a novel method (call DWNN-RLS) to predict new circRNA-disease associations. Firstly, DWNN-RLS computes the Gaussian interaction profile (GIP) kernel similarities of circRNAs and diseases based on the known circRNA-disease associations. By considering their direct acyclic graph(DAG) representation, the sematic similarity of diseases is also calculated. We further obtain the final similarity of diseases with the mean of GIP similarity and sematic similarity. Then the association possibility scores of circRNA-disease pairs are predicted by Kronecker product kernel based Regularized Least Squares approach. The kernels of circRNA-disease pairs are calculated by the Kronecker product of kernels of circRNAs and diseases. Furthermore, the decreasing weight k-nearest neighbor (DWNN) method is used to calculate the initial relational scores of new circRNAs and new diseases. In order to assess the prediction performance of DWNN-RLS and compare with other competing methods, we conduct 5-fold cross validation (5CV), 10-fold cross validation (10CV) and leave-one-out cross validation (LOOCV). The experiment results demonstrate that DWNN-RLS outperforms other six competing methods (RLS-avg, RLS-Kron, NetLapRLS, KATZ, NBI, WP) in terms of AUC (area under the ROC curve) values. Specifically, the AUC values of DWNN-RLS in 5CV, 10CV and LOOCV reach 0.8854, 0.9205 and 0.9701, respectively, which are superior to the second best results (KATZ: 0.8224 and 0.8343, RLS-avg: 0.9169). Furthermore, the prediction ability of DWNN-RLS also is illustrated by the case studies.

## Methods

### Materials

In this study, we download the known circRNA-disease associations data from the CircR2Disease database (http://bioinfo.snnu.edu.cn/CircR2Disease/). These circRNA-disease associations were curated circRNA-disease associations from the existing literature prior to 31 March 2018. After removing the duplicated data, we obtain the benchmark dataset that includes 725 circRNA-disease associations, 676 circRNAs and 100 diseases. In addition, the Mesh database [37] (https://www.nlm.nih.gov/bsd/disted/meshtutorial/themeshdatabase/) is used to compute the sematic similarity of diseases.

### Similarity of circRNAs

As the successful application of GIP kernel similarity in other relative areas [38–42], we also use it to calculate the similarities of circRNAs. The GIP kernel was computed from the known circRNA-disease associations. Let $C=\left \{c_{1},c_{2},...,c_{N_{c}}\right \}$ be the set of *N*_*c*_ circRNAs and $D=\left \{d_{1},d_{2},...,d_{N_{d}}\right \}$ be the set of *N*_*d*_ diseases. Let matrix $\phantom {\dot {i}\!}Y \in R^{N_{c} \times N_{d}}$ represents known circRNA-disease associations, in which the value of *y*_*ij*_ is 1 if circRNA *i* and disease *j* exists a known association, otherwise 0. Then the GIP similarity of circRNA *c*_*i*_ and circRNA *c*_*j*_ can be computed as follows:

1 $$\begin{array}{@{}rcl@{}} S_{c}\left(c_{i},c_{j}\right)= G_{c}\left(c_{i},c_{j}\right) = exp\left(-\gamma_{c} {||y_{c_{i}}-y_{c_{j}}||}^{2}\right)  \end{array} $$

2 $$  \gamma_{c} = 1 /\left(\frac{1}{N_{c}}\sum\limits_{i=1}^{N_{c}}{||y_{c_{i}}||}^{2}\right),  $$

where $y_{c_{i}}=\left \{y_{i1},y_{i2},...,y_{{i}{N_{d}}}\right \}$ and $y_{c_{j}}=\left \{y_{j1},y_{j2},...,y_{{j}{N_{d}}}\right \}$ are the association profiles of circRNA *c*_*i*_ and circRNA *c*_*j*_, respectively. Since the GIP kernel is computed by a decaying function of the distance between the vectors, this function is of the form of a bell-shaped curve. In addition, since a larger value of *γ*_*c*_ yields a narrower bell while a smaller value of *γ*_*c*_ yields a wider bell, the parameter *γ*_*c*_ can be used to regulate the bandwidth of kernel. In this study, parameter *γ*_*c*_ is computed as the reciprocal of average number of associations per circRNA.

### Similarity of diseases

Firstly, we also compute the GIP similarity of disease *d*_*i*_ and disease *d*_*j*_ as follows:

3 $$\begin{array}{@{}rcl@{}} G_{d}\left(d_{i},d_{j}\right) = exp\left(-\gamma_{d} {||y_{d_{i}}-y_{d_{j}}||}^{2}\right)  \end{array} $$

4 $$  \gamma_{d} = 1 /\left(\frac{1}{N_{d}}\sum\limits_{i=1}^{N_{d}}{||y_{d_{i}}||}^{2}\right),  $$

where $y_{d_{i}}=\left \{y_{1i},y_{2i},...,y_{{N_{c}}{i}}\right \}^{T}$ is the association profiles of disease *d*_*i*_ while $y_{d_{j}}=\left \{y_{1j},y_{2j},...,y_{{N_{c}}{j}}\right \}^{T}$ is the association profiles of disease *d*_*j*_. In addition, the parameter *γ*_*d*_ is used to regulate the bandwidth of kernel.

Secondly, we use the Mesh descriptions of diseases to compute the sematic similarity. Specifically, for disease A which can be represented by a DAG (*DAG*_*A*_,*DAG*_*A*_=*T*_*A*_,*E*_*A*_) in mesh database. Set *T*_*A*_ includes the parent diseases nodes of *A* and itself while set *E*_*A*_ includes the direct edges between disease nodes within *T*_*A*_. The similarity of diseases *A* and *B* can be calculated as follows:

5 $$\begin{array}{@{}rcl@{}}  {D_{semsim}(A,B)} = \frac{\sum\limits_{t \in {T_{A}}\cap{T_{B}}}\left({SV}_{A}(t)+{SV}_{B}(t)\right)}{Sem(A)+Sem(B)}, \end{array} $$

where *SV*_*A*_(*t*)(*SV*_*B*_(*t*)) is the sematic value between disease *A*(*B*) and *t* which is the all common ancestors of diseases *A* and *B*. In addition, *Sem*(*A*) and *Sem*(*B*) are the sematic values of diseases *A* and *B*, respectively. For disease *A*, the *Sem*(*A*) and *SV*_*A*_(*t*) can be calculated as follows:

6 $$\begin{array}{@{}rcl@{}}  {Sem(A)} = {\sum\limits_{t \in {T_{A}}}{SV}_{A}(t)}, \end{array} $$

7 $$\begin{array}{@{}rcl@{}}  {{SV}_{A}(t)} \,=\,\! \left\{ \begin{aligned} \!\!1&, t=A \\ \Delta^{w} &, t=\!the~ smallest~ w~ layer\ ancestor~ node~ of~ A \\ \end{aligned} \right. \end{array} $$

where *Δ* is the layer contribution factor between disease node and its direct ancestor disease nodes in DAG. The value of *Δ* is set to 0.5 in this study [37].

After computing the GIP similarity and sematic similarity of diseases, we integrate the final similarity of diseases with their mean as follows:

8 $$\begin{array}{@{}rcl@{}}  S_{d} = \frac{G_{d}+D_{semsim}}{2}, \end{array} $$

### DWNN for new circRNAs and diseases

The good performance of prediction method largely depends on the quality of known circRNA-disease associations. In fact, new circRNAs (or new diseases) have no any association with diseases (or circRNAs). In this study, we use the DMNN to compute the initial association score based on similarities of circRNAs and diseases. Specifically, the initial association score between new circRNA *c*_*i*_ and disease *d*_*j*_ can be calculated as follows:

9 $$ y\left(c_{i},d_{j}\right) = \frac{\sum G{^{il}_{c}}y_{lj}}{\sum G{^{il}_{c}}}, c_{l} \in N{(c_{i})}   $$

where *N*(*c*_*i*_) is the set of $k_{c_{i}}$ nearest neighbors of new circRNA *c*_*i*_. The parameter $k_{c_{i}}$ is calculated as follows:

10 $$\begin{array}{@{}rcl@{}}  {k_{c_{i}}} = \left\{ \begin{aligned} max(k)&,if \ \frac{1-simset(c_{i})_{l}}{l}\le \epsilon{^{l}}\,1\le l \le k \\ 0&,otherwise \\ \end{aligned} \right. \end{array} $$

where *simset*(*c*_*i*_)_*l*_ is the l-th similarity value of the ranked vector based on similarity between circRNA *c*_*i*_ and other circRNAs from high to low. Furthermore, the parameter *ε* is used to control the range of *ε*^*l*^ that is used to select *k* nearest neighbors for each new circRNA and disease. In this study, the value of *ε* is set to 1, so the value of *ε*^*l*^ is 1 and all neighbors are used to calculate initial association score.

Similarly, we also compute the initial association scores of new disease *d*_*j*_ and circRNA *c*_*i*_ as follows:

11 $$ y\left(c_{i},d_{j}\right) = \frac{\sum G{^{jl}_{d}}y_{il}}{\sum G{^{jl}_{d}}}, d_{l} \in N{(d_{j})}   $$

where *N*(*d*_*j*_) is the set of $k_{d_{j}}$ nearest neighbors of new disease *d*_*j*_. The parameter $k_{d_{j}}$ is also calculated as follows:

12 $$\begin{array}{@{}rcl@{}}  {k_{d_{j}}} = \left\{ \begin{aligned} max(k)&,if \ \frac{1-simset(d_{j})_{l}}{l}\le \epsilon{^{l}}\,1\le l \le k \\ 0&,otherwise \\ \end{aligned} \right. \end{array} $$

where *simset*(*d*_*j*_)_*l*_ is the l-th similarity value of the ranked vector based on similarity between disease *d*_*j*_ and other diseases from high to low. Parameter *ε* is also used to control the range for selecting neighbors.

### Kronecker product kernel based regularized least squares(RLS-Kron)

In this study, we use RLS-Kron method to predict new circRNA-disease associations [38, 39, 43]. Based on the kernel *K*, the predicted circRNA-disease associations matrix has a simple closed-form solution as follows:

13 $$ vec\left({\hat Y}^{T}\right) = K{\left(K+\sigma I\right)}^{-1}vec\left(Y^{T}\right)   $$

in which the parameter *σ* is a regularizations parameter and is set to 0.2 in this study. Kron-RLS has no any prediction ability when *σ* is set to 0. The kernel *K* is calculated from the Kronecker product *K*_*c*_⊗*K*_*d*_ of the circRNA kernel and disease kernel, which is defined as follows:

14 $$ K\left(\left(c_{i},d_{j}\right),\left(c_{u},d_{v}\right)\right) = K_{c}(d_{i},d_{u})K_{d}(t_{j},t_{v})   $$

where matrices *K*_*c*_ and *K*_*d*_ are the similarity matrices of circRNAs and diseases, respectively. In addition, in order to calculate the predicted matrix, Kron-RLS needs to compute the inverse of an *N*_*c*_*N*_*d*_×*N*_*c*_*N*_*d*_ matrix. Therefore, we also use an effective method based on matrix eigenvalue decomposition. According to the matrix theory, the eigenvalues (vectors) of a kronecker product are the Kronecker product of eigenvalues (vectors). Specifically, the kernal can be calculated as follows:

15 $$ K = K_{c} \otimes K_{d} = \vee \wedge {\vee}^{T}   $$

where ∧=∧_*c*_⊗∧_*d*_ and ∨=∨_*c*_⊗∨_*d*_ are all derived from the eigenvalues decompositions of the two kernel matrices *K*_*c*_ and *K*_*d*_. As *K*_*c*_ and *K*_*d*_ are real symmetric matrices, their specific eigenvalues decompositions process are defined as follows:

16 $$ K_{c}={\vee}_{c}{\wedge}_{c}{\vee}{_{c}^{T}}   $$

17 $$  K_{d}={\vee}_{d}{\wedge}_{d}{\vee}{_{d}^{T}}  $$

where ∨_*c*_ and ∨_*d*_ are orthogonal matrices whose columns are the eigenvectors of *K*_*c*_ and *K*_*d*_, respectively. ∧_*c*_ and ∧_*d*_ are diagonal matrices whose diagonal entries are the eigenvalues of *K*_*c*_ and *K*_*d*_, respectively. Therefore, the final predicted circRNA-disease associations matrix ${\hat Y}$ can be calculated as follows:

18 $$\begin{array}{@{}rcl@{}} {\hat Y} = {\vee}_{c}{Z^{T}}{\vee}{_{d}^{T}}  \end{array} $$

19 $$ vec(Z) =({\wedge}_{c} \otimes {\wedge}_{d})({\wedge}_{c} \otimes {\wedge}_{d}+ \sigma I)^{-1}vec\left({\vee}{_{d}^{T}}Y^{T}{\vee}{_{c}}\right)  $$

## Results

### Performance evaluation

In this study, we conduct 5CV, 10CV and LOOCV to evaluate the performance of DWNN-RLS for predicting new circRNA-disease associations. AUC (area under the ROC curve) value is used as the evaluation metric.

We perform 10 repetitions of 10CV and 5CV. That is, under 10CV, the known circRNA-disease associations data are divided into 10 folds, and each fold takes in turn as the test set and the rest as the train set at each time. Similarly, the data set are randomly divided into 5 folds and each fold takes in turn as the test data and the rest as the train set on each time. In LOOCV, each known circRNA-disease association is in turn chosen as the test set while the rest known circRNA -disease associations as the train set. The larger AUC values show the better prediction ability of the method, while if AUC value is less than or equal to 0.5, the prediction method has no prediction ability.

### Comparison with other methods

As there is no competing computational method for predicting circRNA-disease associations in the literature, to assess the performance of our method, we also compare DWNN-RLS against other six effective methods in other relevant prediction issues. These methods include RLS-avg [38], RLS-Kron [38], NetLapRLS [44], KATZ [45, 46], NBI [47] and WP [47, 48]. We briefly review them here. RLS-avg use the average of the output values which are computed from two kernels, respectively. RLS-Kron compute the prediction scores by Kronecker product kernel based on regularised least squares approach. NetLapRLS is used to predict circRNA-disease associations by exploiting information on similarities of links and nodes. KATZ is a network-based method which considers the number of walks between network nodes and lengths in a heterogeneous network to predict associations. NBI is also a network-based method to infer new associations, which only uses cricRNA-disease bipartite network topology similarity. WP and DBSI are recommendation models which directly use the similarities of circRNAs and diseases.

**Fig. 1:**
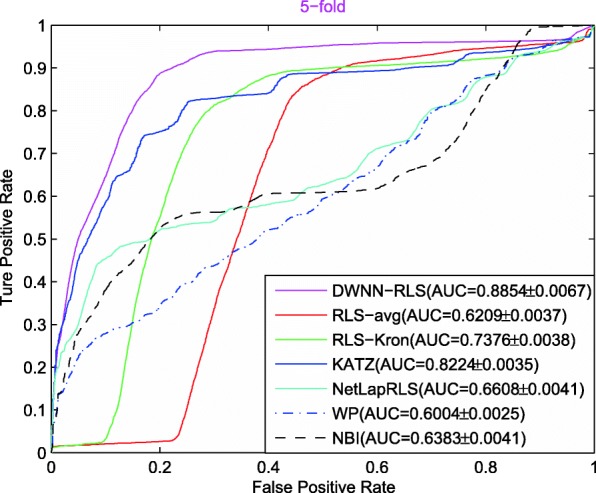
The AUC curves of seven methods in the 5CV

Figure 1 shows the AUC curves of seven prediction methods on CircR2Disease data set in terms of 5CV. The AUC value of DWWN-RLS is the highest among the seven methods, indicating that the prediction performance of DWWN-RLS is better than other methods.

**Fig. 2:**
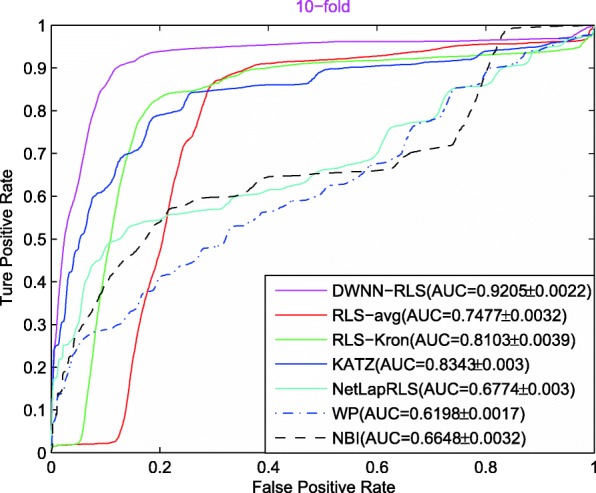
The AUC curves of seven methods in the 10CV

Figure 2 shows the AUC curves of seven prediction methods in terms of 10CV on CircR2Disease dataset. The AUC value of DWWN-RLS reaches 0.9205, which is better than other methods (RLS-avg: 0.7477, RLS-Kron: 0.8103, NetLapRLS: 0.6744, KATZ: 0.8343, NBI: 0.6648, WP: 0.6198).

**Fig. 3:**
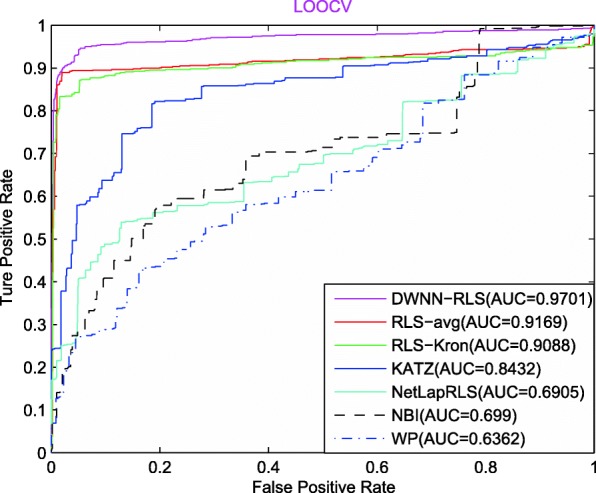
The AUC curves of seven methods in the LOOCV

Figure 3 shows the prediction comparison result between DWWN-RLS and other six methods in terms of LOOCV on CircR2Disease data set. We can see from the Fig.3 that the prediction performance of DWWN-RLS (0.9701) is superior to other methods in terms of AUC values (RLS-avg: 0.9169, RLS-Kron: 0.9088, NetLapRLS: 0.6905, KATZ: 0.8432, NBI: 0.699, WP: 0.6362).

Note that the advantage of prediction performance is more obvious in 10CV and LOOCV than 5CV, indicating that DWWN-RLS can achieve good result based on more known circRNA-disease associations. In addition, the sematic similarity of diseases can improve the prediction performance of DWWN-RLS. When only the GIP similarity is used, the AUCs of DWNN-RLS are 0.8368, 0.8819 and 0.9423 in 5CV, 10CV and LOOCV, respectively. When the GIP similarity combined with the disease sematic similarity, DWNN-RLS obtains the increased AUCs of 0.8854, 0.9205 and 0.9701 in 5CV, 10CV and LOOCV. By comparing with RLS-Kron method, the DMNN method also can improve the prediction performance. Comparing with KATZ, NBI and WP methods, we think that DWNN-RLS is a machining learning model and has the objective function and solution process that is beneficial to obtain better prediction performance.

### Parameter analysis for *ε* and *σ*

To further understand the robustness of DWWN-RLS method, we analyze the influence of parameters *ε* and *σ* on the prediction performance in 10CV. The parameter *ε* is used to control the range for selecting *k* nearest neighbors of cicrRNAs and diseases. The parameter *σ* is the regularization parameter of DWWN-RLS method. The value of *ε* is set to be 1.0 when analyzing parameter *σ*. Furthermore, we also set the default value of *σ* to be 0.2 when analyzing parameter *ε*. With parameter *σ* of 0.2, Table 1 demonstrates the prediction performance of DWWN-RLS method in 10CV when *ε* ranges from 0.1 to 1.0 with 0.1 increments. The prediction performance of DWWN-RLS method is best when *ε* is set to be 1.0, indicating that all neighbors of circRNAs and diseases are involved in calculating their initial associations scores.

**Table 1:** The 10CV prediction performance of various parameter values of *ε* ranging from 0.1 to 1.0 with 0.1 increments, the best result is in bold face

*ε*	0.1	0.2	0.3	0.4	0.5
AUC	0.7927 ±0.0048	0.7927 ±0.0035	0.7922 ±0.0042	0.7902 ±0.0034	0.7920 ±0.0035
*ε*	0.6	0.7	0.8	0.9	1.0
AUC	0.7922 ±0.0042	0.7889 ±0.0032	0.7903 ±0.0047	0.7897 ±0.0044	**0.9205 ±0.0022**

Furthermore, Table 2 describes the prediction performances of DWWN-RLS with different values of *σ* when *ε* is set to be 1.0. We can see from Table 2 that DWWN-RLS obtains the best prediction performance when *σ* is set to be 0.2. Therefore, in this study, we set the default value of *σ* to be 0.2.

**Table 2:** The 10CV prediction performance of various parameter values of *σ* ranging from 0.1 to 1.0 with 0.1 increments, the best result is in bold face

*σ*	0.1	0.2	0.3	0.4	0.5
AUC	0.9200 ±0.0024	**0.9205 ±0.0022**	0.9182 ±0.0023	0.9154 ±0.0018	0.9136 ±0.0021
*σ*	0.6	0.7	0.8	0.9	1.0
AUC	0.9110 ±0.0025	0.9078 ±0.0033	0.9042 ±0.0020	0.9041 ±0.0020	0.9010 ±0.0025

### Case studies

After confirming the prediction performance and robustness of DWWN-RLS method in 10CV, 5CV and LOOCV, we further analyze the prediction ability of DWWN-RLS in discovering new circRNA-disease associations. In predicting new circRNA-disease associations, all known circRNA-disease associations on CircR2Disease dataset are chosen as the train set and all other circRNA-disease pairs are the candidate circRNA-disease associations. We adapt DWWN-RLS to compute the prediction scores for these candidate circRNA-disease pairs. Here, we analyze the prediction results of Atherosclerotic vascular disease and Breast cancer.

Atherosclerotic vascular disease is responsible for the majority of cases of CVD (Cardiovascular disease) in both developing and developed countries, which encompasses coronary heart disease, cerebrovascular disease, and peripheral arterial disease, and which also result the CVD, the leading cause of death and disability all over the world [49, 50]. Table 3 shows that 2 of top 10 predicted associations are confirmed in the previous literature. Elevated cANRIL expression could lead to worse EC (endothelial cells) inflammation, exacerbating AS (atherosclerosis) [51]. CANRIL is transcribed at a locus of atherosclerotic cardiovascular disease on chromosome 9p21, and induces nucleolar stress and apoptosis, and inhibits the proliferation in smooth muscle cells and macrophages [52]. The cZNF292 also associates with atherosclerotic cardiovascular disease by stimulating angiogenesis through vascular sprouting and cell proliferation [53].

**Table 3:** The validation results of predicted top 10 new circRNA-disease associations of Atherosclerotic vascular disease

Disease	CircRNA	Rank	Source
Atherosclerotic vascular disease	cANRIL	1	PMID:28683453,
			PMID:28946214
	hsa_circ_0003575	2	Unknown
	circSMARCA5/hsa_circ_0001445	3	Unknown
	hsa_circ_0000284/circHIPK3	4	Unknown
	hsa_circ_0004383/cZNF292	5	PMID:27836747
	circRNA-chr19	6	Unknown
	CircDOCK1/hsa_circ_100721	7	Unknown
	mmu-circRNA-015947	8	Unknown
	hsa-circRNA 2149	9	Unknown
	circRar1	10	Unknown

There is approximately 1 in 12 women developing breast cancer in Western Europe and the United States, and which is characterized by a distinct metastatic pattern involving the regional lymph nodes, bone marrow, lung and liver [54, 55]. Table 4 shows the validation results of top 10 new circRNA-disease associations predicted by DWNN-RLS. There is 3 out of top 10 predicted associations that can be validated in previous studies. CircRNAs circGFRA1 and GFRA1 act as ceRNAs in triple negative breast cancer by regulating miR-34a [56]. The human breast cancer cell line MDA-MB-231 are stably transfected with circ-Foxo3, the ectopic expression of the Foxo3 circular RNA could suppress tumor growth, cancer cell proliferation and survival [25]. CDR1as contains more than 70 selectively conserved target sites of miR-7 which can directly downregulate oncogenes in cancers such as breast cancer [57].

**Table 4:** The validation results of predicted top 10 new circRNA-disease associations of Breast cancer

Disease	CircRNA	Rank	Source
Breast cancer	circGFRA1/hsa_circ_005239	1	PMID:29037220
	circUBAP2	2	Unknown
	circ-Foxo3/hsa_circ_0006404	3	PMID:26657152
	Cir-ITCH/hsa_circ_0001141/hsa_circ_001763	4	Unknown
	hsa_circ_0001649	5	Unknown
	CDR1as/ciRS-7/hsa_circ_0001946	6	PMID:28049499
	hsa_circ_0043256	7	Unknown
	hsa_circ_0016760	8	Unknown
	hsa_circ_0007385	9	Unknown
	hsa_circ_0014130	10	Unknown

Above case studies demonstrate that there are a number of prediction results that have not been confirmed by previous literature. To our knowledge, a possible reason is that the database Circ2Disease are still limited and the new studies have not been published yet. In summary, these predicted circRNA-disease associations deserve being studied and considered in the future.

## Discussion

With the advances of RNA-Seq, high-throughput sequencing and other techniques, we have achieved some important progresses in understanding characteristics and functions of cricRNAs. CricRNAs may play key roles in diseases as miRNA sponges or decoys, protein sponges or decoys and regulation gene transcription. Therefore, systematically understanding association between circRNAs and diseases has become an important issue of bioinformatics research, which is beneficial to disease diagnose and treatment. Although some databases about circRNA have been established in recent years, these databases rarely focused on the associations between circRNAs and diseases. The computation methods for predicting circRNA-disease associations are also lacking because of these limitations. To our knowledge, CircR2Disease is the first database about circRNA-disease associations, which provides the chance to develop effective methods to identify novel associations between circRNAs and diseases.

## Conclusion

DWNN-RLS method is developed to predict new associations between circRNAs and diseases on CircR2Disease dataset. Firstly, DWNN-RLS computes the GIP similarities of circRNAs and diseases based on the known circRNA-disease associations. Secondly, we further compute the sematic similarity of disease and compute the final similarity of diseases with the mean of GIP similarity and sematic similarity. Finally, the Kron-RLS is used to predict novel circRNA-disease associations based on their similarities. 10CV, 5CV and LOOCV are used to evaluate the prediction performance of DWNN-RLS. In addition, we use the DWNN to calculate the initial associations scores for new circRNAs and diseases. We also compare our method with other six methods. In terms of 10CV, 5CV and LOOCV, DWNN-RLS all achieves the best prediction performance. In addition, we also show that DWNN-RLS method may achieves better prediction performance with the more known circRNA-disease associations. Case studies further illustrate the prediction performance of DWNN-RLS.

However, there still exist some limitations in DWNN-RLS. We all know that cricRNAs can function as miRNA sponges or decoys, protein sponges or decoys. In this study, we only use the GIP similarity of circRNAs. In the future, the similarity computation of circRNAs could consider more relevant biological network information, such as cricRNA-miRNA associations and sequence information. Similarly, the disease functional information also should be considered [58–60]. Other latest matrix factorization methods such as NRLMF [61], SRMF [62], DRRS [63] should be considered to predict cricRNA-disease association when we integrate more biological network information such as circRNA-miRNA associations, circRNA sequence information and disease functional information. Therefore, to further improve the prediction performance, we would develop a more effective approach to discover new circRNA-disease associations by reasonably integrating more biological network information.

## References

[1] Nigro JM, Cho KR, Fearon ER, Kern SE, Ruppert JM, Oliner JD, Kinzler KW, Vogelstein B (1991). Scrambled exons. Cell.

[2] Zhang Y, Zhang X-O, Chen T, Xiang J-F, Yin Q-F, Xing Y-H, Zhu S, Yang L, Chen L-L (2013). Circular intronic long noncoding rnas. Mol Cell.

[3] Knupp D, Miura P (2018). Circrna accumulation: A new hallmark of aging?. Mech Ageing Dev.

[4] Memczak S, Jens M, Elefsinioti A, Torti F, Krueger J, Rybak A, Maier L, Mackowiak SD, Gregersen LH, Munschauer M (2013). Circular rnas are a large class of animal rnas with regulatory potency. Nature.

[5] Jeck WR, Sorrentino JA, Wang K, Slevin MK, Burd CE, Liu J, Marzluff WF, Sharpless NE (2013). Circular rnas are abundant, conserved, and associated with alu repeats. Rna.

[6] Enuka Y, Lauriola M, Feldman ME, Sas-Chen A, Ulitsky I, Yarden Y (2015). Circular rnas are long-lived and display only minimal early alterations in response to a growth factor. Nucleic Acids Res.

[7] Cocquerelle C, Mascrez B, Hetuin D, Bailleul B (1993). Mis-splicing yields circular rna molecules. FASEB J.

[8] Lasda E, Parker R (2014). Circular rnas: diversity of form and function. Rna.

[9] Ye C-Y, Chen L, Liu C, Zhu Q-H, Fan L (2015). Widespread noncoding circular rnas in plants. New Phytol.

[10] Kristensen L, Hansen T, Venø M, Kjems J (2018). Circular rnas in cancer: opportunities and challenges in the field. Oncogene.

[11] Danan M, Schwartz S, Edelheit S, Sorek R (2011). Transcriptome-wide discovery of circular rnas in archaea. Nucleic Acids Res.

[12] Chu Q, Zhang X, Zhu X, Liu C, Mao L, Ye C, Zhu Q-H, Fan L (2017). Plantcircbase: a database for plant circular rnas. Mol Plant.

[13] Legnini I, Di Timoteo G, Rossi F, Morlando M, Briganti F, Sthandier O, Fatica A, Santini T, Andronache A, Wade M (2017). Circ-znf609 is a circular rna that can be translated and functions in myogenesis. Mol Cell.

[14] Qu S, Yang X, Li X, Wang J, Gao Y, Shang R, Sun W, Dou K, Li H (2015). Circular rna: a new star of noncoding rnas. Cancer Lett.

[15] Ashwal-Fluss R, Meyer M, Pamudurti NR, Ivanov A, Bartok O, Hanan M, Evantal N, Memczak S, Rajewsky N, Kadener S (2014). circrna biogenesis competes with pre-mrna splicing. Mol Cell.

[16] Du WW, Fang L, Yang W, Wu N, Awan FM, Yang Z, Yang BB (2017). Induction of tumor apoptosis through a circular rna enhancing foxo3 activity. Cell Death Differ.

[17] Li Z, Huang C, Bao C, Chen L, Lin M, Wang X, Zhong G, Yu B, Hu W, Dai L (2015). Exon-intron circular rnas regulate transcription in the nucleus. Nat Struct Mol Biol.

[18] Ghosal S, Das S, Sen R, Basak P, Chakrabarti J (2013). Circ2traits: a comprehensive database for circular rna potentially associated with disease and traits. Front Genet.

[19] Li J, Zheng Y, Sun G, Xiong S (2014). Restoration of mir-7 expression suppresses the growth of lewis lung cancer cells by modulating epidermal growth factor receptor signaling. Oncol Rep.

[20] Li F, Zhang L, Li W, Deng J, Zheng J, An M, Lu J, Zhou Y (2015). Circular rna itch has inhibitory effect on escc by suppressing the wnt/ *β*-catenin pathway. Oncotarget.

[21] Anastas JN, Moon RT (2013). Wnt signalling pathways as therapeutic targets in cancer. Nat Rev Cancer.

[22] Hansen TB, Jensen TI, Clausen BH, Bramsen JB, Finsen B, Damgaard CK, Kjems J (2013). Natural rna circles function as efficient microrna sponges. Nature.

[23] Wang Q, Tang H, Yin S, Dong C (2013). Downregulation of microrna-138 enhances the proliferation, migration and invasion of cholangiocarcinoma cells through the upregulation of rhoc/p-erk/mmp-2/mmp-9. Oncol Rep.

[24] Zhong Z, Huang M, Lv M, He Y, Duan C, Zhang L, Chen J (2017). Circular rna mylk as a competing endogenous rna promotes bladder cancer progression through modulating vegfa/vegfr2 signaling pathway. Cancer Lett.

[25] Yang W, Du W, Li X, Yee A, Yang B (2016). Foxo3 activity promoted by non-coding effects of circular rna and foxo3 pseudogene in the inhibition of tumor growth and angiogenesis. Oncogene.

[26] Han D, Li J, Wang H, Su X, Hou J, Gu Y, Qian C, Lin Y, Liu X, Huang M (2017). Circular rna mto1 acts as the sponge of mir-9 to suppress hepatocellular carcinoma progression. Hepatology.

[27] Hsiao K-Y, Lin Y-C, Gupta SK, Chang N, Yen L, Sun HS, Tsai S-J (2017). Noncoding effects of circular rna ccdc66 promote colon cancer growth and metastasis. Cancer Res.

[28] Glažar P, Papavasileiou P, Rajewsky N (2014). circbase: a database for circular rnas. Rna.

[29] Chen X, Han P, Zhou T, Guo X, Song X, Li Y (2016). circrnadb: a comprehensive database for human circular rnas with protein-coding annotations. Sci Rep.

[30] Zhang P, Meng X, Chen H, Liu Y, Xue J, Zhou Y, Chen M (2017). Plantcircnet: a database for plant circrna–mirna–mrna regulatory networks.. Database.

[31] Li S, Li Y, Chen B, Zhao J, Yu S, Tang Y, Zheng Q, Li Y, Wang P, He X (2017). exorbase: a database of circrna, lncrna and mrna in human blood exosomes. Nucleic Acids Res.

[32] Liu Y-C, Li J-R, Sun C-H, Andrews E, Chao R-F, Lin F-M, Weng S-L, Hsu S-D, Huang C-C, Cheng C (2015). Circnet: a database of circular rnas derived from transcriptome sequencing data. Nucleic Acids Res.

[33] Xia S, Feng J, Lei L, Hu J, Xia L, Wang J, Xiang Y, Liu L, Zhong S, Han L (2016). Comprehensive characterization of tissue-specific circular rnas in the human and mouse genomes. Brief Bioinform.

[34] Bhattacharya A, Cui Y (2015). Somamir 2.0: a database of cancer somatic mutations altering microrna–cerna interactions. Nucleic Acids Res.

[35] Xia S, Feng J, Chen K, Ma Y, Gong J, Cai F, Jin Y, Gao Y, Xia L, Chang H (2017). Cscd: a database for cancer-specific circular rnas. Nucleic Acids Res.

[36] Fan C, Lei X, Fang Z, Jiang Q, Wu F-X (2018). Circr2disease: a manually curated database for experimentally supported circular rnas associated with various diseases. Database.

[37] Wang D, Wang J, Lu M, Song F, Cui Q (2010). Inferring the human microrna functional similarity and functional network based on microrna-associated diseases. Bioinformatics.

[38] van Laarhoven T, Nabuurs SB, Marchiori E (2011). Gaussian interaction profile kernels for predicting drug–target interaction. Bioinformatics.

[39] Yan C, Wang J, Lan W, Wu F-X, Pan Y (2017). Sdtrls: Predicting drug-target interactions for complex diseases based on chemical substructures. Complexity.

[40] Yan C, Wang J, Ni P, Lan W, Wu FX, Pan Y. Dnrlmf-mda:predicting microrna-disease associations based on similarities of micrornas and diseases. IEEE/ACM Trans Comput Bio Bioinforma. 2017(to be published). 10.1109/TCBB.2017.2776101. 29990253

[41] Lan W, Li M, Zhao K, Liu J, Wu F-X, Pan Y, Wang J (2016). Ldap: a web server for lncrna-disease association prediction. Bioinformatics.

[42] Lu C, Yang M, Luo F, Wu F-X, Li M, Pan Y, Li Y, Wang J (2018). Prediction of lncrna-disease associations based on inductive matrix completion. Bioinformatics.

[43] Raymond R, Kashima H (2010). Fast and scalable algorithms for semi-supervised link prediction on static and dynamic graphs. Joint European Conference on Machine Learning and Knowledge Discovery in Databases.

[44] Xia Z, Wu LY, Zhou X (2010). Semi-supervised drug-protein interaction prediction from heterogeneous biological spaces. BMC Syst Biol.

[45] Chen X, Huang Y-A, You Z-H, Yan G-Y, Wang X-S (2016). A novel approach based on katz measure to predict associations of human microbiota with non-infectious diseases. Bioinformatics.

[46] Qu Y, Zhang H, Liang C, Dong X (2018). Katzmda: prediction of mirna-disease associations based on katz model. IEEE Access.

[47] Cheng F, Liu C, Jiang J, Lu W, Li W, Liu G, Zhou W, Huang J, Tang Y (2012). Prediction of drug-target interactions and drug repositioning via network-based inference. PLoS Comput Biol.

[48] Yamanishi Y, Araki M, Gutteridge A, Honda W, Kanehisa M (2008). Prediction of drug–target interaction networks from the integration of chemical and genomic spaces. Bioinformatics.

[49] Organization WH. The World Health Report 2002. http://www.who.int/whr/en. Accessed 24 Aug 2018.

[50] Hackam DG, Anand SS (2003). Emerging risk factors for atherosclerotic vascular disease: a critical review of the evidence. Jama.

[51] Song C-L, Wang J-P, Xue X, Liu N, Zhang X-H, Zhao Z, Liu J-G, Zhang C-P, Piao Z-H, Liu Y (2017). Effect of circular anril on the inflammatory response of vascular endothelial cells in a rat model of coronary atherosclerosis. Cell Physiol Biochem.

[52] Li C-Y, Ma L, Yu B (2017). Circular rna hsa_circ_0003575 regulates oxldl induced vascular endothelial cells proliferation and angiogenesis. Biomed Pharmacother.

[53] Devaux Y (2017). Transcriptome of blood cells as a reservoir of cardiovascular biomarkers. Biochim Biophys Acta (BBA) - Mol Cell Res.

[54] Wooster R, Bignell G, Lancaster J, Swift S, Seal S, Mangion J, Collins N, Gregory S, Gumbs C, Micklem G (1995). Identification of the breast cancer susceptibility gene brca2. Nature.

[55] Müller A, Homey B, Soto H, Ge N, Catron D, Buchanan ME, McClanahan T, Murphy E, Yuan W, Wagner SN (2001). Involvement of chemokine receptors in breast cancer metastasis. nature.

[56] He R, Liu P, Xie X, Zhou Y, Liao Q, Xiong W, Li X, Li G, Zeng Z, Tang H (2017). circgfra1 and gfra1 act as cernas in triple negative breast cancer by regulating mir-34a. J Exp Clin Cancer Res.

[57] Dong Y, He D, Peng Z, Peng W, Shi W, Wang J, Li B, Zhang C, Duan C (2017). Circular rnas in cancer: an emerging key player. J Hematol Oncol.

[58] Cheng L, Li J, Ju P, Peng J, Wang Y (2014). Semfunsim: a new method for measuring disease similarity by integrating semantic and gene functional association. PloS ONE.

[59] Lan W, Wang J, Li M, Liu J, Wu F-X, Pan Y. Predicting microrna-disease associations based on improved microrna and disease similaritiesOncol. IEEE/ACM Trans Comput Biol Bioinforma. 2016(to be published). 10.1109/TCBB.2016.2586190. 27392365

[60] Ni P, Wang J, Zhong P, Li Y, Wu F, Pan Y. Constructing disease similarity networks based on disease module theory. IEEE/ACM Trans Comput Biol Bioinforma. 2018(to be published). 10.1109/TCBB.2018.2817624. 29993782

[61] Liu Y, Wu M, Miao C, Zhao P, Li X. -L. (2016). Neighborhood regularized logistic matrix factorization for drug-target interaction prediction. PLoS computational biology.

[62] Wang L, Li X, Zhang L, Gao Q (2017). Improved anticancer drug response prediction in cell lines using matrix factorization with similarity regularization. BMC cancer.

[63] Luo H, Li M, Wang S, Liu Q, Li Y., Wang J. (2018). Computational drug repositioning using low-rank matrix approximation and randomized algorithms. Bioinformatics.

